# Circulating FGF21 and GDF15 as Biomarkers for Screening, Diagnosis, and Severity Assessment of Primary Mitochondrial Disorders in Children

**DOI:** 10.3389/fped.2022.851534

**Published:** 2022-04-14

**Authors:** Yi Li, Shengrui Li, Yinfeng Qiu, Maobin Zhou, Min Chen, Yue Hu, Siqi Hong, Li Jiang, Yi Guo

**Affiliations:** ^1^Department of Neurology, Children's Hospital of Chongqing Medical University, Chongqing, China; ^2^National Clinical Research Center for Child Health and Disorders, Chongqing, China; ^3^Ministry of Education Key Laboratory of Child Development and Disorders, Chongqing, China; ^4^Chongqing Key Laboratory of Pediatrics, Chongqing, China

**Keywords:** primary mitochondrial disorders, childhood, GDF15, FGF21, biomarkers

## Abstract

**Background:**

Primary mitochondrial disorders (PMDs) are a diagnostic challenge for paediatricians, and identification of reliable and easily measurable biomarkers has become a high priority. This study aimed to investigate the role of serum fibroblast growth factor 21 (FGF21) and growth differentiation factor 15 (GDF15) in children with PMDs.

**Methods:**

We analysed serum FGF21 and GDF15 concentrations by enzyme-linked immunosorbent assay (ELISA) in children with PMDs, patients with non-mitochondrial neuromuscular disorders (NMDs), and aged-matched healthy children, and compared them with serum lactate and ratio of lactate and pyruvate (L/P). We also evaluated correlations between these biomarkers and the phenotype, genotype, and severity of PMDs.

**Results:**

The median serum GDF15 and FGF21 concentrations were significantly elevated in fifty-one patients with PMDs (919.46 pg/ml and 281.3 pg/ml) compared with those of thirty patients with NMDs (294.86 pg/ml and 140.51 pg/ml, both *P* < 0.05) and fifty healthy controls (221.21 pg/ml and 85.02 pg/ml, both *P* < 0.05). The area under the curve of GDF15 for the diagnosis of PMDs was 0.891, which was higher than that of the other biomarkers, including FGF21 (0.814), lactate (0.863) and L/P ratio (0.671). Calculated by the maximum Youden index, the critical value of GDF15 was 606.369 pg/ml, and corresponding sensitivity and specificity were 74.5and 100%. In the PMD group, FGF21 was significantly correlated with International Paediatric Mitochondrial Disease Scale (IPMDS) score. The levels of GDF15 and FGF21 were positively correlated with age, critical illness condition, and multisystem involvement but were not correlated with syndromic/non-syndromic PMDs, different mitochondrial syndromes, nuclear DNA/mitochondrial DNA pathogenic variants, gene functions, or different organ/system involvement.

**Conclusion:**

Regardless of clinical phenotype and genotype, circulating GDF15 and FGF21 are reliable biomarkers for children with PMDs. GDF15 can serve as a screening biomarker for diagnosis, and FGF21 can serve as a severity biomarker for monitoring.

## Introduction

Primary mitochondrial disorders (PMDs) are the most common inherited metabolic disorders caused by pathogenic variants in either mitochondrial DNA (mtDNA) or nuclear DNA (nDNA), and to impaired mitochondrial function, which is characterised by defects in oxidative phosphorylation (1, 2). PMDs are highly notoriously heterogeneous in clinical manifestations, with involvement of any organ system at any age and presentation (3). To date, disease-causing variants have been identified in over 300 genes implicated in mitochondrial metabolism (4). Because PMDs have high clinical and genetic heterogeneity, their diagnosis often presents a challenge even for experienced clinicians, which is particularly true in children (5).

Analysis of metabolites (e.g., lactate, pyruvate, alanine, acylcarnitine, creatine, and organic acids) in the blood, urine, and cerebrospinal fluid (CSF) may help in providing a global picture and raising the suspicion of PMDs (6). However, these biomarkers are too non-specific for PMDs and do not provide significant help in many cases, possibly because the muscles, myocardium, and brain are not affected in every patient with PMDs or may be unaffected at the time of investigation, and some of these parameters depend on whether these fluids are collected at rest, during exercise or with a proper sampling method (7–9). Muscle biopsy and enzymatic activity analysis of respiratory chain complexes are not always available, and, in some cases, enzyme activity is difficult to interpret. Next-generation sequencing (NGS) technology is also not suitable for large-scale disease screening because of its high price and long detection period. For these reasons, the exploration of more reliable and easily measurable biomarkers is ongoing.

Recent studies have found that serum fibroblast growth factor 21 (FGF21) and growth differentiation factor 15 (GDF15) appear to be promising biomarkers for identifying PMDs (10–12). FGF21 is a hormone-like cytokine that is involved in the intermediary metabolism of carbohydrates and lipids (13). Since 2011, FGF21 has been repeatedly reported as a potential biomarker for mitochondrial myopathies and PMDs caused by mechanisms primarily or secondarily affecting mitochondrial translation, such as direct mutation of a translation machinery and mtDNA deletion (10, 13–15). GDF15 serves as a TGF-β family protein that is produced upon the inflammation and oxidative stress to maintain tissue homeostasis (16, 17). The role of GDF15 as a biomarker for PMDs has been tested on large cohorts of patients with different mitochondrial defects, and has shown its higher sensitivity and specificity than FGF21 (6, 12, 18). Furthermore, GDF15 levels were higher in patients with PMDs and TK2 defects or multisystem involvement and PMDs due to mitochondrial translation and mtDNA maintenance defects (11, 12). However, most studies were on adult cohorts and had controversial results (6, 11–21). For example, some authors reported that FGF21 and GDF15 were correlated with PMD severity and could be used as reliable indexes of disease progression, but Koene et al. showed that the two biomarkers were not correlated with disease severity in a large cohort of adult m.3243A>G mutation carriers (20, 21). Furthermore, FGF21 and GDF15 have been reported to be elevated in a range of non-mitochondrial disorders, including cancers, obesity, renal diseases, diabetes, and liver diseases (22, 23). The utility of FGF21 and GDF15 as biomarkers in children with PMDs still needs to be determined in broader, well-characterised mitochondrial cohorts. In this study, we investigated FGF21 and GDF15 levels in a cohort of children and evaluated the significance of FGF21 and GDF15 levels in the diagnosis, severity assessment, and correlation of phenotypes and genotypes of PMDs.

## Materials and Methods

### Study Population

Consecutive patients with PMDs and non-mitochondrial disorders (NMDs) were enrolled from the neurology department of Children's Hospital of Chongqing Medical University from 2015 to 2019. Patients with PMDs fulfilled the clinicopathological and/or genetic criteria of PMDs (24, 25). Patients were diagnosed with NMDs according to their standard criteria. For the healthy controls, we recruited age- and sex-matched healthy individuals from the health physical examination center of the Children's Hospital of Chongqing Medical University. This study was approved by the ethics committee of the Children's Hospital affiliated to Chongqing Medical University, and written informed consent was obtained from all the participants.

### Plasma Collection and FGF21 and GDF15 Concentration Quantification

Fasting peripheral blood was collected from patients with PMDs and those with NMDs in the morning of the day after admission. The blood samples were collected in 10-ml lithium heparin tubes and then centrifuged at 1,500 rpm/min for 10 min. The supernatant (i.e., plasma) was collected in 1-ml conical vials and frozen at −80°C as soon as possible. We used a commercially available enzyme-linked immunosorbent assay kit to measure plasma FGF21 and GDF15 concentrations in accordance with the manufacturer's instructions (Finetest, CN).

### Clinical Data

Clinical data, including clinical presentations, genetic data, and serum concentrations of lactate and pyruvic acid, were retrospectively collected for all the patients.

The International Paediatric Mitochondrial Disease Scale (IPMDS) was used to assess the severity and natural history of patients with PMDs on the day after admission. Two doctors performed the scoring process independently to avoid the influence of subjective factors on score results. The average score was obtained when the gap in the score was <5. Disagreements were resolved by discussion with a third investigator. Higher scores indicate worse conditions (26).

### Statistical Analyses

Recorded data were analysed using the SPSS 26.0 and Medcalc 19.4.0 software. Quantitative data were expressed as mean ± standard deviation (SD). Qualitative data were expressed as frequency and percentage. Independent samples *t*-test of significance was performed when comparing two means. Correlations between different subgroups and GDF15 and FGF21 levels were analysed by Spear-man's correlation test. Receiver operating characteristic (ROC) curve analysis was performed to find the best cut-off value with detection of sensitivity and specificity at this cut-off value. *P*-value < 0.05 was considered significant.

## Results

### Patient Characteristics

A total of 131 samples from 51 patients with PMDs (age range 11–96 months, median age 36 months, M:F = 1:0.7), 30 patients with NMDs (age range 34.8–84 months, median age 41.5 months, M:F = 1:0.88), and 50 healthy children (age range 21–58.3 months, median age 37 months, M:F = 1:1) were analysed. In the PMD group, subgroups were divided according to clinical and genetic information, including syndromic/non-syndromic PMDs, different syndromes, nDNA/mtDNA pathogenic variants, gene function, and different organ/system involvement. In the NMD group, there were seven patients with Duchenne muscular dystrophy (DMD), six patients with myasthenia gravis, 15 patients with epilepsy, and two patients with viral encephalitis (Supplementary Tables 1, 2).

In the PMD group, the levels of GDF15, FGF21 and two other traditional biomarkers (lactate and lactate/pyruvate (L/P) ratio) were significantly increased compared with those in the NMD group and the healthy control group. The median serum GDF15 concentration in the patients with PMDs was 919.46 pg/ml compared to the 294.86 pg/ml in the patients with NMDs and 221.21 pg/ml in the healthy controls. The median serum GDF15 concentration in the patients with PMDs was 3.12 times higher than that in the patients with NMDs and 4.2 times higher than that in the healthy controls. The median serum FGF21 concentrations of the three groups were 281.30, 140.51, and 85.02 pg/ml, respectively. The median serum FGF21 level in the patients with PMDs was 1.99 times higher than that in the patients with NMDs and 3.3 times higher than that in the healthy controls.

In the NMD group, the levels of circulatory FGF21 and GDF15 were slightly to moderately increased (Table 1). Nineteen patients had serum FGF21 levels above the upper limit (116.591 pg/ml), including 10 patients with epilepsy (7/15, 46.7%), 5 patients with myasthenia gravis (5/6, 83.3%), 3 patients with DMD (3/7, 42.9%), and 1 patient with viral encephalitis (1/2, 50%). Among them, the patients with myasthenia gravis and the one with viral encephalitis showed only slightly elevation, and those with epilepsy and DMD had more obvious elevations. Serum GDF15 was elevated in 15 patients (upper limit 297.568 pg/ml), including 8 patients with epilepsy (8/15, 53%), 3 patients with myasthenia gravis (3/6, 50%), 3 patients with DMD (3/7, 42.9%), and 1 patient with viral encephalitis (1/2, 50%). However, the elevated levels of GDF15 and FGF21 in this group were not significantly different compared with those in the healthy control group.

**Table 1:** Values of biomarkers in the different groups (95% CI).

**Biomarkers**	**PMDs**	**NMDs**	**Healthy control**
MedianFGF21 (pg/ml)	281.30 (142.16–569.96)***	140.51 (54.48–226.06)*	85.02 (53.87–131.82)
MedianGDF15 (pg/ml)	919.46 (539.45–1670.27)***	294.86 (139.01–448.0)	221.21 (117.09–360.64)
Median Lactate (mmol/l)	4.24 (2.72–7.29)***	2.09 (1.05–3.09)	1.65 (0.82–2.45)
Median L/P ratio	9.38 (9.63–18.72)**	7.28 (4.15–11.07)	6.40 (4.23–9.48)

*, P < 0.05; **, P < 0.01; ***, P < 0.001.

### Association of Serum Levels of GDF15 and FGF21 With the Clinical/Genetic Characteristics of PMDs Patients

We evaluated the correlations between GDF15 and FGF21 and the phenotype and genotype of PMDs. The results revealed that in the critical illness condition (ICU admission) and multiorgan/system (≥2 systems) involvement groups, the levels of GDF15 and FGF21 were also increased significantly compared with those of the groups without these conditions. Unfortunately, the levels of GDF15 and FGF21 were not significantly different in the skeletal muscle/nervous/digestive tract/kidney/cardiac/respiratory system involvement, different PMD syndromes, syndromic/non-syndromic PMDs, mtDNA/nDNA, and different gene functional subgroups (Table 2).

**Table 2:** FGF21 and GDF15 in the primary mitochondrial disorder (PMD) subgroups.

**PMDs subgroup classification**		**Patients'**	**GDF15**	**FGF21**
		**number**	**(pg/ml)**	**(pg/ml)**
Nervous system involvement	W/	45	907.10 (591.12, 1,670.27)	281.30 (145.17, 584.25)
	W/O	6	1,006.24 (335.42, 1,395.81)	320.33 (34.68, 595.15)
Skeletal muscle involvement	W/	32	931.32 (387.63, 1,740.04)	401.72 (92.61, 458.61)
	W/O	19	907.10 (757.13, 1,670.27)	529.15 (165.24, 892.61)
Digestive tract involvement	W/	39	943.18 (686.32, 1,670.27)	570.49 (258.80, 2,095.07)
	W/O	12	734.68 (153.45, 1,473.93)	428.19 (103.12, 518.99)
Urinary system involvement	W/	3	489.56 (327.23, 593.45)	275.32 (155.92, 478.39)
	W/O	48	647.48 (492.53, 744.95)	374.04 (297.36, 502.18)
Respiratory system involvement	W/	21	549.32 (397.43, 842.54)	294.65 (197.45-543.56)
	W/O	30	626.98 (492.38, 936.54)	385.64 (188.49.673.27)
Cardiacinvolvement	W/	32	759.43(496.28, 1,003.26)	295.39 (224.76, 543.21)
	W/O	19	684.35 (474.54, 995.43)	392.18 (266.48-630.72)
ICU admission	W/	31	979.11 (757.13, 1,829.28)*	481.30 (142.16, 598.55)*
	W/O	20	578.11 (164.49, 1,280.77)	209.12 (116.14, 539.10)
Multi-organ/system involvement	W/	43	943.18 (539.45, 1,670.27)*	570.49 (325.45, 860.60)*
	W/O	8	477.12 (154.65, 1,395.16)	231.18 (103.12, 529.15)
Syndromic PMDs		46	532.45 (178.45, 1,909.04)	439.27 (116.64, 3.394.87)
	MELAS	22	1,011.15 (740.93, 1,909.04)	307.61 (146.68, 585.03)
	Leigh syndrome	16	813.37 (533.14, 1,464.49)	170.26 (116.64, 528.09)
	MERRF	2	612.255	408.39
	Combinedoxidative phosphorylation defect	2	1,092.28	468.79
	Mitochondrial DNA depletion	2	1,264.92	127.66
	Primary Q10 deficiency	1	2,100.21	3,394.87
	Barth syndrome	1	1,037.336	753.342
Non- Syndromic PMDs		5	186.32 (129.67, 1,374.09)	254.69 (54.16, 1,871.34)
Genotype	mtDNA	35	975.14 (491.37, 1,670.27)	319.21 (157.21, 569.96)
	nDNA	15	816.38 (539.45, 1,472.34)	165.24 (91.11, 753.34)
Genetic functional type	Translation machinery	27	943.18 (491.37,1,670.27)	296.96 (157.21, 569.96)
	RC subunits/assembly	21	816.38 (518.02, 1,641.87)	172.26 (97.12, 661.26)
	Homeostasis	1	1,037.34	753.34
	Inhibitor	1	887.44	165.24

W/, with; W/O, without; *, P < 0.05; **, P < 0.01; ***, P < 0.001.

### Diagnostic Performance of GDF15 and FGF21

**Figure 1:**
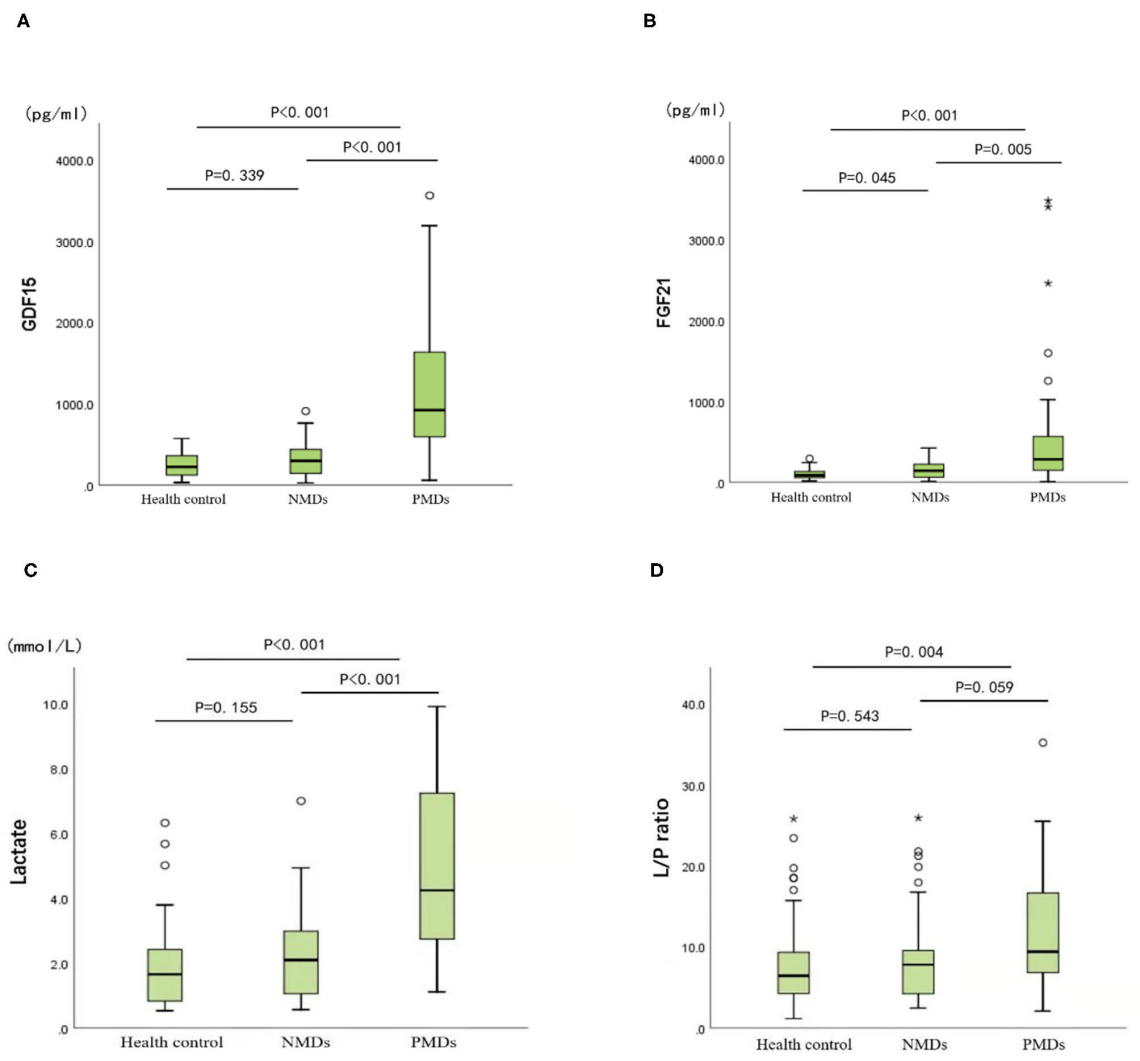
Biomarker concentrations in the healthy controls, mitochondrial diseases, and other neuromuscular diseases. **(A)** GDF15. **(B)** FGF21. **(C)** Lactate. **(D)** Lactate/pyruvate. Mann-Whitney U non-parametric test is performed for comparison between groups. *P < 0.05, **P < 0.01.

As shown in Figure 1, unlike other serum biomarkers, the reference range of GDF15 concentration in PMDs patients was markedly narrow, which exhibited little overlap with that of patients with NMDs or healthy controls. Furthermore, there was no difference in GDF15 or FGF21 concentrations between the patients with NMDs and the healthy controls.

The cut-off value was defined as the upper limit of normal [95% confidence interval (CI)] of each biomarker in the healthy control group. The cut-off value of FGF21 was 116.591 pg/ml, that of GDF15 was 297.568 pg/ml, that of lactic acid was 2.924 mmol/L, and that of L/P ratio was 15.826. The sensitivity of GDF15 in diagnosing PMDs was better than that of FGF21 and lactate, and specificity was slightly worse than that of the latter two. FGF21 performed similar specificity to lactate but was less sensitive than lactate (Table 3). The diagnostic odds ratios of FGF21, GDF15, lactate, and L/P ratio in the patients with PMDs were 8.375, 11.256, 10.899, and 2.601, respectively.

**Table 3:** Sensitivity (SE), specificity (SP), positive predictive value (PPV), and negative predictive value (NPV) of each biomarker. (95% CI).

**Biomarkers**	**SE%**	**SP%**	**PPV%**	**NPV%**
FGF21	76.47 (62.18–86.75)	72.00 (57.29–83.33)	73.58 (59.42–84.32)	75.00 (60.11–85.89)
GDF15	86.27 (73.13–93.85)	62.00 (47.16–75.00)	69.84 (56.82–80.43)	81.58 (65.11–91.68)
Lactate	82.35 (68.64–91.13)	70.00 (55.22–81.71)	73.68 (60.09–84.06)	79.55 (64.25–89.67)
L/P ratio	45.01 (31.38–59.55)	76.00 (61.51–86.48)	65.71 (47.74–80.32)	57.58 (44.82–69.45)

**Figure 2:**
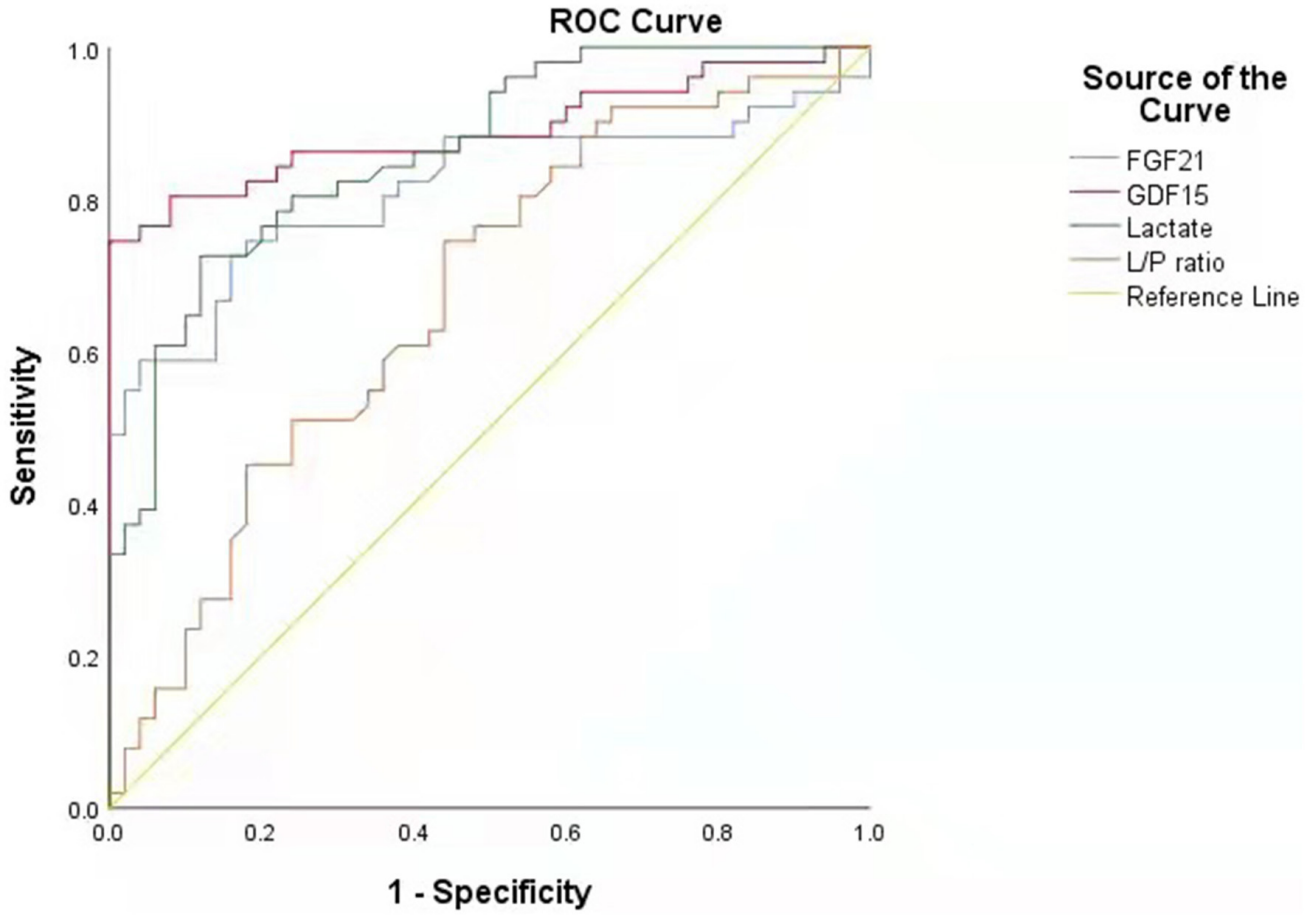
Receiver operating characteristic (ROC) curve of each biomarker in diagnosis of PMDs.

Performing receiver operating characteristic (ROC) curve analyses (Figure 2), GDF15 was identified as the best indicator of PMDs considering the combination of sensitivity and specificity (area under the curve, AUC, = 0.891; 95% CI.822~0.959) compared with the other biomarkers, including FGF21 (AUC = 0.814; 95% CI.725~0.902), lactate (AUC = 0.863; 95% CI.794~0.932), and L/P ratio (AUC = 0.671; 95% CI.566~0.776). This result indicates that the chance of correctly identifying PMDs in patients is 89.1% from the measurement of serum GDF15, compared to the 81.4, 86.3, and 67.1% from the measurements of FGF21, lactate, and L/P ratio, respectively. Individual comparisons showed that the AUC, sensitivity, and specificity of GDF15 were significantly higher than those of the other biomarkers, including FGF21 (*P* < 0.05), lactate (*P* < 0.001), and L/P ratio (*P* < 0.001).

Based on the above evaluation of the value of various biomarkers in the diagnosis of PMDs, serum GDF15 has the highest diagnostic value.

### Predictive Value of GDF15 and FGF21 for PMDs

**Figure 3:**
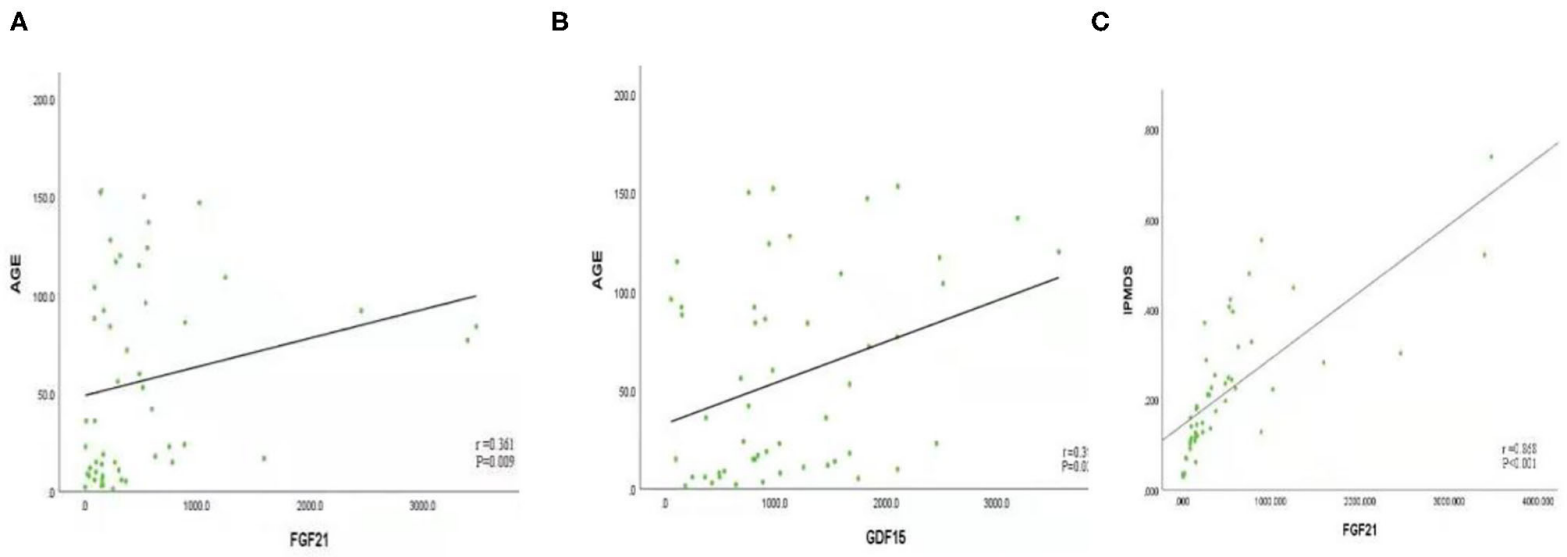
**(A)** FGF21 is positively correlated with age (*r* = 0.361, *P* < 0.05). **(B)** GDF15 is positively correlated with age (*r* = 0.318, *P* < 0.05); **(C)** FGF21 was positively correlated with International Paediatric Mitochondrial Disease Scale (IPMDS) score (*r* = 0.586, *P* < 0.001).

Spearman's rank correlation coefficient was calculated to assess associations among the biomarkers. The concentrations of GDF15 and FGF21 were positively correlated with age (FGF21: *r* = 0.361, *P* < 0.05; GDF15: *r* = 0.318, *P* < 0.05). The concentration of FGF21 was positively correlated with IPMDS score (*r* = 0.586, *P* < 0.001). There was no significant correlation between concentration of GDF15 and IPMDS score (Figure 3, Supplementary Table 3). Furthermore, we did not find any correlation between FGF21 or GDF15 and sex, level of serum lactate, or L/P ratio.

## Discussion

This study discusses the role of serum FGF21 and GDF15 with a cohort of children with PMDs. Our study is different from most previous studies in this field, since those studies are mainly focused on adults or a single phenotype/genotype. We focused exclusively on children, and we evaluated the correlations between the two biomarkers and various phenotypes, genotypes, and severity of PMDs.

This study revealed that GDF15 is superior to FGF21, lactate, and L/P ratio in sensitivity, specificity, diagnostic odds ratio value, and AUC value, suggesting that GDF15 has the best diagnostic value in screening for diagnosis of children with PMDs, which is consistent with the findings of previous studies (6, 11, 12, 27, 28). In addition, our study found that the range of serum GDF15 fluctuations in children with PMDs did not overlap with those in the disease control group and the healthy control group, suggesting that GDF15 can serve as the most suitable screening diagnostic index for children with PMDs.

In addition, we found that in the PMD group, FGF21 was also significantly elevated, however,compared with GDF15 and lactic acid, it did not have the advantages of specificity and sensitivity, therefore, FGF21 was not the first choice as a screening indicator. However, this study found a significant correlation between FGF21 concentration and IPMDS score of the patients with PMDs. It is well-known that the IPMDS not only allows for the clinical status of patients with mitochondrial disease to be evaluated over time but also facilitates the assessment of patients' quality of life. This scale is specific for patients with PMDs and has been shown to be a valid, consistent tool with good reproducibility (26). From this result, we believe that FGF21 can serve as a potential predictor of the severity of PMDs in children. In other words, higher level of FGF21 corresponds to greater mitochondrial disease severity (higher IPMDS scores).

We evaluated the correlations between FGF21 and GDF15 and various phenotypes/genotypes, including syndromic/non-syndromic PMDs, different mitochondrial syndromes (MELAS, Leigh syndrome, etc.), nDNA/mtDNA pathogenic variants, gene functions, and different organ/system involvement. First, we found that the concentrations of GDF15 and FGF21 were positively correlated with age; that is, GDF15 and FGF21 increase during the ageing process, which was similar to the findings of other recent publications (28, 29). Therefore, it may be necessary to formulate different diagnostic cut-off values of GDF15 and FGF21 for adults and children. FGF21 was first reported as a potential biomarker in PMDs, since its serum concentration was found sensitive and specific for mitochondrial myopathies (12, 13, 15, 29–31). However, in our cohort, we found that FGF21 was not significantly different between the children with and those without skeletal muscle involvement. We speculate that this may be related to the lower muscle volume in children. In addition, data collection may be missed because of unclear descriptions, lack of cooperation, or parents' inability to observe.

Some previous studies suggested that FGF21 and GDF15 seem to be more specific markers for PMDs due to mitochondrial translation machinery and mtDNA maintenance defects, as opposed to resulting from impaired respiratory chain complexes or assembly factors (32, 33). Some studies found that GDF15 or FGF21 levels were higher in some specific phenotypes of PMDs, for example, in patients with TK2 defects, MELAS, or muscle manifestations (11, 18). However, we did not find any association between GDF15 or FGF21 levels and function of the affected gene, type of mutation (mtDNA/nDNA), PMD syndrome, or type of syndrome. From this perspective, GDF15 and FGF21 seem to be more indicative of PMDs regardless of clinical phenotype and genotype. Of course, this point remains controversial and remains to be tested on broader, larger, and multicentre cohorts.

Human circulating FGF21 is derived mainly from the liver, but the protein is also expressed in adipocytes, myocytes, and the pancreas (15). GDF15 is mainly expressed in the placenta, kidney, liver, lungs, pancreas, and prostate (27). Both play essential roles in regulating cellular response to stress signals, inflammation, and antioxidative pathways (34). In our cohort, a correlation between levels of GDF15 and FGF21 and single organ/system lesions was not found. Moreover, we found that GDF15 and FGF21 were increased most significantly in patients with critical illness and patients with multisystem involvement. These findings demonstrate that the upregulation mechanism of the two biomarkers in PMDs is involved in stress-responsive pathways rather than destruction of target organs. The elevated cytokines act as metabolic mediators of mitochondrial stress adaptation and promote cell metabolism to compensate for the energy insufficiency caused by mitochondrial dysfunction (35).

GDF15 and FGF21 have been found to be associated with a range of non-mitochondrial diseases, including cancer, obesity, renal diseases, diabetes, liver diseases, and non-mitochondrial myopathies (18, 36–38). Therefore, the diagnostic specificity of these biomarkers is still unclear (6). To assess the impact of NMDs, including secondary mitochondrial dysfunction, on the specificity of the two biomarkers, this study examined the GDF15 and FGF21 concentrations in patients with non-mitochondrial neuromuscular disorders, including viral encephalitis, myasthenia gravis, DMD, and epilepsy, with the latter two being frequently associated with secondary mitochondrial dysfunction. Secondary mitochondrial dysfunction is common in a variety of hereditary/non-genetic diseases, including epilepsy, autism spectrum disorder, muscular dystrophy, chromosomal diseases, and severe infections; it plays an important role in the development and occurrence of diseases. Targeted treatment may improve the progression (39). The results revealed that serum FGF21 and GDF15 levels were variably increased in the NMD group, especially in the patients with DMD and epilepsy. However, compared with the PMD group, the increase was not obvious, and compared with the healthy control group, there was also no significant difference. Therefore, our results suggested that, on the one hand, the significant elevation of circulating GDF15 and FGF21 may underlie PMDs, and that on the other hand, mild to moderate increase in GDF15 and FGF21 may suggest suspected secondary mitochondrial dysfunction. Of course, varying elevations in serum FGF21 and GDF15 levels also exist in non-neuromuscular diseases, including diabetes, liver diseases, kidney diseases, tumours, heart diseases, and respiratory diseases (9, 18, 36–38). Our study ruled out the above diseases, but the role of FGF21 and GDF15 in children with non-neuromuscular diseases should be studied in the future.

In conclusion, this study revealed that the levels of circulating GDF15 and FGF21 are helpful non-invasive indices for children with PMDs regardless of phenotype and genotype. GDF15 is superior to previous biomarkers and FGF21 in screening for the diagnosis of PMDs; however, FGF21 may reflect disease severity in children with PMDs. Age and some conditions, including secondary mitochondrial dysfunction, should be taken into account in the determination of results.

## Data Availability Statement

The original contributions presented in the study are included in the article/Supplementary Material, further inquiries can be directed to the corresponding author.

## Ethics Statement

The studies involving human participants were reviewed and approved by Ethics Committee of Children's Hospital Affiliated to Chongqing Medical University. Written informed consent to participate in this study was provided by the participants' legal guardian/next of kin.

## Author Contributions

YG designed the research study. YL performed the research. SL, YQ, and MZ contributed in data collection. MC, YH, SH, and LJ contributed in patient collection. YG and YL wrote the manuscript. All authors contributed to editorial changes in the manuscript, read, and approved the final version of the manuscript.

## Conflict of Interest

The authors declare that the research was conducted in the absence of any commercial or financial relationships that could be construed as a potential conflict of interest.

## Publisher's Note

All claims expressed in this article are solely those of the authors and do not necessarily represent those of their affiliated organizations, or those of the publisher, the editors and the reviewers. Any product that may be evaluated in this article, or claim that may be made by its manufacturer, is not guaranteed or endorsed by the publisher.
