# Supplementary material for: Circulating FGF21 and GDF15 as Biomarkers for Screening, Diagnosis, and Severity Assessment of Primary Mitochondrial Disorders in Children
Source: Front Pediatr. 2022 Apr 14;10:851534. doi: 10.3389/fped.2022.851534 (PMC9047692; doi:10.3389/fped.2022.851534)
Supplement: Supplementary file 1 [file Table_1.DOCX]

**Supplemental Table.1. Phenotype, underlying genetic defects and respective biomarker values in patients with primary mitochondrial**

| No. | Gander | Age (months) | Mitochondrial disease syndrome | | Genetic defects | | Mutation  category | | Median FGF21 (pg/ml) | | Median  GDF15 (pg/ml) | | | Median Lactate (mmol/L) | | Median L/P ratio | |  | |  |
| --- | --- | --- | --- | --- | --- | --- | --- | --- | --- | --- | --- | --- | --- | --- | --- | --- | --- | --- | --- | --- |
| 1 | M | 153 | | MELAS | | m.3243 A>G | | tRNA | | 529.148 | | 759.129 | | | 1.29 | | 5.81 | |  | |
| 2 | F | 100 | | MELAS | | m.3243 A>G | | tRNA | | 379.114 | | 1843.350 | | | 4.21 | | 2.97 | |  | |
| 3 | M | 144 | | MELAS | | m.3243 A>G | | tRNA | | 231.179 | | | 1129.183 | | 9.45 | | 17.7 | |  | |
| 4 | F | 109 | | MELAS | | m.3243 A>G | | tRNA | | 1250.142 | | | 1592.260 | | 1.11 | | 6.99 | |  | |
| 5 | F | 108 | | MERRF | | m.8344 G>A | | tRNA | | 488.459 | | | 975.142 | | 3.26 | | 22.2 | |  | |
| 6 | M | 96 | | nonsyndromic primary mitochondrial disorders | | m.8347 C>T | | tRNA | | 90.113 | | | 157.213 | | 8.30 | | 15.28 | |  | |
| 7 | M | 14 | | Leigh | | m.8994 G>T | | subunits/assembly factors | | 154.205 | | | 1535.170 | | 2.86 | | 7.11 | |  | |
| 8 | M | 84 | | MELAS | | m.3243 A>G | | tRNA | | 228.186 | | | 818.229 | | 8.65 | | 20.91 | |  | |
| 9 | F | 156 | | MELAS | | m.3243 A>G | | tRNA | | 148.189 | | | 2106.120 | | 2.96 | | 17.51 | |  | |
| 10 | F | 115 | | Leigh | | m.8994 G>T | | subunits/assembly factors | | 485.113 | | | 111.124 | | 6.89 | | 7.96 | |  | |
| 11 | F | 164 | | MELAS | | m.3243 A>G | | tRNA | | 142.155 | | | 979.114 | | 8.95 | | 15.04 | |  | |
| 12 | F | 5.3 | | Leigh | | m.8993 T>G | | subunits/assembly factors | | 368.120 | | | 1748.560 | | 2.04 | | 4.15 | |  | |
| 13 | F | 162 | | MELAS | | m.3243 A>G | | tRNA | | 569.958 | | | 3187.112 | | 7.65 | | 15.46 | |  | |
| 14 | M | 19 | | nonsyndromic primary mitochondrial disorders | | m.3169insC | | rRNA | | 271.118 | | | 102.117 | | 8.83 | | 17.88 | |  | |
| 15 | F | 144 | | MELAS | | m.3243 A>G | | tRNA | | 281.295 | | | 2480.114 | | 4.87 | | 13.48 | |  | |
| 16 | F | 151 | | Leigh | | m.11774 A>G | | subunits/assembly factors | | 1019.210 | | | 1829.280 | | 6.79 | | 7.12 | |  | |
| 17 | M | 139 | | MELAS | | m.3243 A>G | | tRNA | | 558.131 | | | 943.177 | | 2.62 | | 8.52 | |  | |
| 18 | F | 120 | | MELAS | | m.3243 A>G | | tRNA | | 319.210 | | | 3557.969 | | 1.62 | | 2.06 | |  | |
| 19 | M | 99 | | nonsyndromic primary mitochondrial disorders | | m.8277-8446del | | subunits/assembly factors | | 3471.560 | | | 1290.207 | | 4.48 | | 7.83 | |  | |
| 20 | F | 197 | | MELAS | | m.3243 A>G | | tRNA | | 92.113 | | | 2514.116 | | 9.37 | | 25.24 | |  | |
| 21 | M | 56 | | Leigh | | m.8346 A>G | | tRNA | | 598.549 | | | 757.132 | | 4.24 | | 5.22 | |  | |
| 22 | M | 113 | | MELAS | | m.3243 A>G | | tRNA | | 892.607 | | | 907.098 | | 8.643 | | 25.49 | |  | |
| 23 | M | 77 | | primary Q10 deficiency | | COQ8A | | subunits/assembly factors | | 3394.870 | | | 2100.207 | | 4.08 | | 7.05 | |  | |
| 24 | M | 39 | | MELAS | | m.3243 A>G | | tRNA | | 630.245 | | | 1670.270 | | 3.91 | | 12.35 | |  | |
| 25 | M | 91 | | MELAS | | m.3243 A>G | | tRNA | | 296.017 | | | 686.316 | | 2.724 | | 5.57 | |  | |
| 26 | M | 24 | | Leigh | | SURF1 | | subunits/assembly factors | | 780.099 | | | 797.098 | | 4.66 | | 13.8 | |  | |
| 27 | M | 28 | | MELAS | | FASTKD2 | | subunits/assembly factors | | 28.186 | | | 539.451 | | 9.91 | | 35.18 | |  | |
| 28 | M | 112 | | MELAS | | m.3243 A>G | | tRNA | | 2453.690 | | | 152.199 | | 4.15 | | 6.63 | |  | |
| 29 | M | 29 | | Combined oxidative phosphorylation deficiency | | GTPBP3 | | subunits/assembly factors | | 887.435 | | | 712.231 | | 4.03 | | 5.24 | |  | |
| 30 | M | 4 | | Leigh | | ECHS1 | | inhibitor | | 165.238 | | | 887.435 | | 2.50 | | 7.27 | |  | |
| 31 | F | 101 | | Leigh | | FASTKD2 | | subunits/assembly factors | | 172.262 | | | 810.367 | | 6.24 | | 14.36 | |  | |
| 32 | F | 4.57 | | Leigh | | m.10158 T>C | | ND3  subunits/assembly factors | | 8.243 | | | 642.780 | | 7.29 | | 5.55 | |  | |
| 33 | M | 63 | | Barth | | TAZ | | Homeostasis | | 753.342 | | | 1037.336 | | 2.3 | | 18.9 | |  | |
| 34 | M | 23 | | Leigh | | NDUFV2 | | subunits/assembly factors | | 12.231 | | | 2453.690 | | 1.505 | | 9.38 | |  | |
| 35 | M | 53 | | MELAS | | m.3243 A>G | | tRNA | | 518.988 | | | 1670.270 | | 4.27 | | 8.349 | |  | |
| 36 | M | 123 | | MELAS | | m.3243 A>G | | tRNA | | 94.113 | | | 374.116 | | 1.59 | | 7.65 | |  | |
| 37 | M | 21 | | Mitochondrial DNA depletion syndrome | | SUCLA2 | | subunits/assembly | | 152.199 | | | 428.186 | | 2.825 | | 25.04 | |  | |
| 38 | M | 144 | | Leigh | | surf1 | | subunits/assembly factors | | 542.421 | | | 57.132 | | 2.89 | | 16.52 | |  | |
| 39 | F | 85 | | Leigh | | m.8347A>G | | tRNA | | 162.240 | | | 491.367 | | 7.19 | | 4.66 | |  | |
| 40 | M | 32 | | Leigh | | GTPBP3 | | subunits/assembly factors | | 91.113 | | | 496.592 | | 8.54 | | 16.72 | |  | |
| 41 | M | 37 | | Leigh | | m.10158T>C | | subunits/assembly factors | | 307.116 | | | 1252.448 | | 3.5 | | 9.542 | |  | |
| 42 | M | 67 | | Leigh | | surf1 | | subunits/assembly factors | | 104.118 | | | 816.375 | | 1.78 | | 6.976 | |  | |
| 43 | F | 101 | | MERRF | | m.8993 T>G | | subunits/assembly factors | | 328.319 | | | 249.367 | | 8.6 | | 19.09 | |  | |
| 44 | M | 51 | | Leigh | | SUCLA2 | | subunits/assembly factors | | 168.248 | | | 919.458 | | 7.79 | | 18.29 | |  | |
| 45 | F | 128 | | MELAS | | m.3243 A>G | | tRNA | | 1592.260 | | | 842.135 | | 5.63 | | 4.962 | |  | |
| 46 | F | 39 | | Combined oxidative phosphorylation deficiency | | GFM1 | | subunits/assembly factors | | 50.142 | | | 1472.338 | | 2.75 | | 6.05 | |  | |
| 47 | M | 42 | | MELAS | | m.3243 A>G | | tRNA | | 157.213 | | | 364.299 | | 1.64 | | 3.846 | |  | |
| 48 | F | 17 | | nonsyndromic primary mitochondrial disorders | | m.769 T>C | | rRNA | | 254.694 | | | 186.316 | | 4.8 | | 7.457 | |  | |
| 49 | M | 123 | | MELAS | | m.3243 A>G | | tRNA | | 42.155 | | | 1043.177 | | 6.34 | | 15.47 | |  | |
| 50 | F | 15 | | Mitochondrial DNA depletion syndrome | | TK2 | | subunits/assembly factors | | 103.117 | | | 2101.654 | | 1.98 | | 15 | |  | |
| 51 | F | 114 | | nonsyndromic primary mitochondrial disorders | | N | | N | | 18.213 | | | 1457.969 | | 5.93 | | 9.58 | |  | |

**Supplemental Table.2. Clinical diagnoses, respective biomarker values in patients with non-mitochondrial** **neuromuscular disorders**

| No. | Gander | Age (months) | Diagnosis | Median FGF21(pg/ml) | Median GDF15(pg/ml) | Median Lactate(mmol/L) | Median  L/P ratio |
| --- | --- | --- | --- | --- | --- | --- | --- |
| 1 | M | 24 | Epilepsy | 39.620 | 148.560 | 4.510 | 19.850 |
| 2 | M | 38 | DMD | 59.950 | 317.250 | 2.611 | 2.430 |
| 3 | M | 84 | DMD | 21.110 | 102.170 | 1.050 | 3.650 |
| 4 | M | 93 | DMD | 29.300 | 480.140 | 4.740 | 4.010 |
| 5 | F | 105 | DMD | 421.650 | 129.580 | 6.997 | 25.900 |
| 6 | F | 57 | Epilepsy | 58.780 | 673.020 | 0.705 | 4.240 |
| 7 | M | 48 | Epilepsy | 394.210 | 407.160 | 1.960 | 7.780 |
| 8 | M | 94 | Viral encephalitis | 41.560 | 430.700 | 1.360 | 8.900 |
| 9 | M | 10 | Epilepsy | 218.420 | 592.590 | 1.040 | 7.950 |
| 10 | F | 18 | Epilepsy | 272.130 | 64.200 | 0.870 | 3.940 |
| 11 | F | 27 | Epilepsy | 25.621 | 79.350 | 1.946 | 8.030 |
| 12 | F | 23 | Epilepsy | 379.490 | 340.270 | 2.270 | 21.200 |
| 13 | M | 59 | Myasthenia gravis | 269.580 | 760.200 | 1.632 | 4.350 |
| 14 | F | 64 | Myasthenia gravis | 71.180 | 214.320 | 0.660 | 8.480 |
| 15 | F | 77 | Myasthenia gravis | 121.295 | 53.760 | 2.260 | 9.100 |
| 16 | F | 83 | Myasthenia gravis | 217.930 | 277.460 | 0.649 | 4.520 |
| 17 | F | 69 | Epilepsy | 218.110 | 907.020 | 3.622 | 16.730 |
| 18 | M | 28 | Epilepsy | 120.210 | 100.380 | 1.850 | 6.920 |
| 19 | F | 54 | Myasthenia gravis | 401.750 | 437.290 | 4.930 | 21.750 |
| 20 | F | 122 | Viral encephalitis | 220.480 | 210.470 | 2.980 | 6.490 |
| 21 | M | 37 | Epilepsy | 192.490 | 512.700 | 2.450 | 7.770 |
| 22 | M | 48 | Epilepsy | 149.452 | 24.190 | 3.830 | 4.190 |
| 23 | M | 54 | DMD | 187.350 | 166.316 | 2.420 | 15.700 |
| 24 | M | 95 | DMD | 10.390 | 142.155 | 1.785 | 3.190 |
| 25 | F | 13 | Epilepsy | 242.780 | 368.120 | 2.910 | 9.530 |
| 26 | F | 64 | Epilepsy | 121.340 | 569.958 | 0.561 | 3.892 |
| 27 | M | 72 | Epilepsy | 107.360 | 271.118 | 2.220 | 6.400 |
| 28 | M | 75 | Epilepsy | 19.210 | 281.295 | 0.907 | 2.440 |
| 29 | M | 84 | Myasthenia gravis | 131.560 | 412.370 | 1.660 | 8.400 |
| 30 | F | 96 | DMD | 172.130 | 308.430 | 3.424 | 17.930 |

**Supplemental Table.3. The score of IPMDS in patients with primary mitochondrial**

| No. | Domain 1 | | Domain 2 | | Domain 3 | | Total score | |
| --- | --- | --- | --- | --- | --- | --- | --- | --- |
|  | raw score | % | raw score | % | raw score | % | raw score | % |
| 1 | 22/74 | 29.73% | 9/57 | 15.79% | 38/39 | 97.44% | 69/170 | 40.59% |
| 2 | 27/103 | 26.21% | 5/67 | 7.46% | 8/59 | 13.56% | 40/229 | 17.47% |
| 3 | 22/98 | 22.45% | 1/61 | 1.64% | 1/29 | 3.45% | 24/188 | 12.77% |
| 4 | 42/84 | 50.00% | 8/57 | 14.04% | 42/64 | 65.63% | 92/205 | 44.88% |
| 5 | 9/103 | 8.74% | 4/61 | 6.56% | 28/44 | 63.64% | 41/208 | 19.71% |
| 6 | 12/98 | 12.24% | 1/61 | 1.64% | 6/49 | 12.24% | 19/208 | 9.13% |
| 7 | 11/84 | 13.10% | 0/61 | 0.00% | 0/34 | 0.00% | 11/179 | 6.15% |
| 8 | 11/79 | 13.92% | 6/57 | 10.53% | 9/39 | 23.08% | 26/175 | 14.86% |
| 9 | 8/98 | 8.16% | 2/61 | 3.28% | 12/29 | 41.38% | 22/188 | 11.70% |
| 10 | 13/55 | 23.64% | 3/57 | 5.26% | 14/15 | 93.33% | 30/127 | 23.62% |
| 11 | 19/84 | 22.62% | 0/61 | 0.00% | 2/49 | 4.08% | 21/194 | 10.82% |
| 12 | 31/98 | 31.63% | 11/67 | 16.42% | 15/59 | 25.42% | 57/224 | 25.45% |
| 13 | 30/76 | 39.47% | 3/52 | 5.77% | 37/49 | 75.51% | 70/177 | 39.55% |
| 14 | 14/88 | 15.91% | 13/57 | 22.81% | 26/39 | 66.67% | 53/184 | 28.80% |
| 15 | 8/84 | 9.52% | 11/61 | 18.03% | 21/44 | 47.73% | 40/189 | 21.16% |
| 16 | 20/98 | 20.41% | 8/67 | 11.94% | 22/59 | 37.29% | 50/224 | 22.32% |
| 17 | 13/84 | 15.48% | 5/57 | 8.77% | 26/39 | 66.67% | 44/180 | 24.44% |
| 18 | 14/74 | 18.92% | 3/61 | 4.92% | 6/34 | 17.65% | 23/169 | 13.61% |
| 19 | 56/74 | 75.68% | 31/63 | 49.21% | 58/59 | 98.31% | 145/196 | 73.98% |
| 20 | 6/103 | 5.83% | 4/61 | 6.56% | 13/49 | 26.53% | 23/213 | 10.80% |
| 21 | 18/94 | 19.15% | 7/61 | 11.48% | 21/49 | 42.86% | 46/204 | 22.55% |
| 22 | 31/69 | 44.93% | 17/47 | 36.17% | 38/39 | 97.44% | 86/155 | 55.48% |
| 23 | 30/65 | 46.15% | 16/57 | 28.07% | 38/39 | 97.44% | 84/161 | 52.17% |
| 24 | 31/89 | 34.83% | 6/61 | 9.84% | 23/39 | 58.97% | 60/189 | 31.75% |
| 25 | 21/93 | 22.58% | 13/67 | 19.40% | 12/59 | 20.34% | 46/219 | 21.00% |
| 26 | 37/93 | 39.78% | 14/67 | 20.90% | 21/59 | 35.59% | 72/219 | 32.88% |
| 27 | 5/74 | 6.76% | 0/57 | 0.00% | 1/34 | 2.94% | 6/165 | 3.64% |
| 28 | 7/69 | 10.14% | 3/47 | 6.38% | 37/39 | 94.87% | 47/155 | 30.32% |
| 29 | 18/84 | 21.43% | 4/61 | 6.56% | 1/34 | 2.94% | 23/179 | 12.85% |
| 30 | 19/79 | 24.05% | 4/62 | 6.45% | 3/39 | 7.69% | 26/180 | 14.44% |
| 31 | 16/89 | 17.98% | 1/61 | 1.64% | 5/34 | 14.71% | 22/184 | 11.96% |
| 32 | 6/98 | 6.12% | 0/61 | 0.00% | 1/39 | 2.56% | 7/198 | 3.54% |
| 33 | 25/79 | 31.65% | 13/57 | 22.81% | 58/64 | 90.63% | 96/200 | 48.00% |
| 34 | 2/79 | 2.53% | 3/61 | 4.92% | 0/34 | 0.00% | 5/174 | 2.87% |
| 35 | 24/83 | 28.92% | 12/67 | 17.91% | 16/59 | 27.12% | 52/209 | 24.88% |
| 36 | 14/103 | 13.59% | 5/61 | 8.20% | 15/49 | 30.61% | 34/213 | 15.96% |
| 37 | 15/103 | 14.56% | 12/67 | 17.91% | 2/59 | 3.39% | 29/229 | 12.66% |
| 38 | 27/84 | 32.14% | 6/26 | 23.08% | 30/39 | 76.92% | 63/149 | 42.28% |
| 39 | 27/93 | 29.03% | 0/57 | 0.00% | 8/39 | 20.51% | 35/189 | 18.52% |
| 40 | 14/98 | 14.29% | 3/61 | 4.92% | 2/29 | 6.90% | 19/188 | 10.11% |
| 41 | 27/88 | 30.68% | 9/67 | 13.43% | 9/59 | 15.25% | 45/214 | 21.03% |
| 42 | 12/83 | 14.46% | 2/67 | 2.99% | 10/59 | 16.95% | 24/209 | 11.48% |
| 43 | 29/103 | 28.16% | 14/63 | 22.22% | 8/59 | 13.56% | 51/225 | 22.67% |
| 44 | 18/98 | 18.37% | 9/67 | 13.43% | 14/59 | 23.73% | 41/224 | 18.30% |
| 45 | 36/74 | 48.65% | 8/57 | 14.04% | 4/39 | 10.26% | 48/170 | 28.24% |
| 46 | 10/88 | 11.36% | 2/61 | 3.28% | 0/24 | 0.00% | 12/173 | 6.94% |
| 47 | 19/98 | 19.39% | 4/67 | 5.97% | 17/59 | 28.81% | 40/224 | 17.86% |
| 48 | 29/93 | 31.18% | 6/61 | 9.84% | 44/59 | 74.58% | 79/213 | 37.09% |
| 49 | 10/93 | 10.75% | 2/61 | 3.28% | 1/29 | 3.45% | 13/183 | 7.10% |
| 50 | 12/79 | 15.19% | 2/57 | 3.51% | 10/34 | 29.41% | 24/170 | 14.12% |
| 51 | 3/103 | 2.91% | 3/67 | 4.48% | 2/59 | 3.39% | 8/229 | 3.49% |
